# EGF receptor lysosomal degradation is delayed in the cells stimulated with EGF-Quantum dot bioconjugate but earlier key events of endocytic degradative pathway are similar to that of native EGF

**DOI:** 10.18632/oncotarget.17873

**Published:** 2017-05-15

**Authors:** Anna V. Salova, Tatiana N. Belyaeva, Ekaterina A. Leontieva, Elena S. Kornilova

**Affiliations:** ^1^ Institute of Cytology of the Russian Academy of Sciences, St. Petersburg, Russia; ^2^ Peter the Great St. Petersburg Polytechnic University, St. Petersburg, Russia; ^3^ St. Petersburg State University, St. Petersburg, Russia; ^4^ ITMO University, St. Petersburg, Russia

**Keywords:** EGF receptor, quantum dots, endocytosis dynamics, lysosomes

## Abstract

Quantum dots (QDs) complexed to ligands recognizing surface receptors undergoing internalization are an attractive tool for live cell imaging of ligand-receptor complexes behavior and for specific tracking of the cells of interest. However, conjugation of quasi-multivalent large QD-particle to monovalent small growth factors like EGF that bound their tyrosine-kinase receptors may affect key endocytic events tightly bound to signaling. Here, by means of confocal microscopy we have addressed the key endocytic events of lysosomal degradative pathway stimulated by native EGF or EGF-QD bioconjugate. We have demonstrated that the decrease in endosome number, increase in mean endosome integrated density and the pattern of EEA1 co-localization with EGF-EGFR complexes at early stages of endocytosis were similar for the both native and QD-conjugated ligands. In both cases enlarged hollow endosomes appeared after wortmannin treatment. This indicates that early endosomal fusions and their maturation proceed similar for both ligands. EGF-QD and native EGF similarly accumulated in juxtanuclear region, and live cell imaging of endosome motion revealed the behavior described elsewhere for microtubule-facilitated motility. Finally, EGF-QD and the receptor were found in lysosomes. However, degradation of receptor part of QD-EGF-EGFR-complex was delayed compared to native EGF, but not inhibited, while QDs fluorescence was detected in lysosomes even after 24 hours. Importantly, in HeLa and A549 cells the both ligands behaved similarly. We conclude that during endocytosis EGF-QD behaves as a neutral marker for degradative pathway up to lysosomal stage and can also be used as a long-term cell marker.

## INTRODUCTION

Quantum dots (QDs) are fluorescent semiconductor nanoparticles with a high quantum yield and exceptional photostability [1–4]. Their complexing to ligands recognizing surface receptors that undergo internalization makes them an attractive tool for live cell imaging of ligand-receptor complexes behavior and for specific delivery of fluorescent probes into the cells of interest [5–10]. However, functionalized QDs are much larger than native ligands and due to their large surface become quasi-multivalent. Evidently, concern about a possible influence of QDs on the behavior of ligand-receptor complexes could not be excluded especially since some side effects have been reported [11–15]. This problem becomes increasingly important when a QD is complexed to monovalent growth factors that bind their specific receptors at 1-to-1 ratio like EGF and must then form dimers to be internalized and trigger proper intracellular signaling. Taking into account a tight cross-talk between endocytosis and signaling, it is possible to suggest that any deviations in key endocytic events may manifest changes in signaling process. Unfortunately, this problem has not been extensively investigated.

In this paper we used the EGF-EGF receptor (EGFR) model to compare the effects of QD-labeled EGF on the key events during the entire course of endocytosis, from early stages up to lysosomal degradation, with those of a native ligand. The choice of EGF-EGFR is explained by several reasons. First, the EGF receptor, a member of c-ErbB family, is a typical representative of signaling tyrosine kinases (TK) [16–20]; second, it is the most studied one among numerous receptor TK families [21–24], which allows a valid comparison of many parameters of endocytosis, stimulated by EGF and EGF labeled with QDs. Finally, abnormalities in its functioning – overexpression or expression of certain mutant forms – are tightly bound to numerous epithelial malignancies including carcinomas of breast, lung, esophagus, head and neck [25–30].

According to current concept, EGF binds to its receptor with high affinity (10^–9^–10^–11^ M). Formation of EGF:EGFR dimers stimulates endocytosis through clathrin-coated pits on the plasma membrane. Coated vesicles are then pinched off by the atypical GTPase dynamin [31]. Activation of the EGF receptor TK indirectly leads to the recruitment of small GTPase Rab5 to the newly formed endosomes. Rab5, being in its active GTP-bound form, organizes the subsequent events that finally result in the degradation of both the ligand and receptor in the lysosomes [32–34]. The main event triggered by Rab5 is recruiting of phosphatidylinositol-3-kinase Vps34, which forms domains enriched in PI-3-monophosphate (PI3P) on the endosomal membrane. This lipid is recognized by FYVE-domain-containing proteins. Active Rab5 and PI3P are both necessary for the binding of the so-called early endosomal autoantigen EEA1, which works as a tethering factor in homotypic fusion of early endosomes, allowing them to enlarge. Then, endosomal membrane invaginations begin to form within these domains and further turn into the internal vesicles of late multivesicular endosomes (MVE) with the help of ESCRT0-IV protein complexes, some components of which possess FYVE-domains. Simultaneously, ESCRT complexes provide concentrating of cargo proteins there and their translocation into internal vesicles of MVE, thus preventing recycling. And then, the cargoes became available for degradation by lysosomal enzymes within the hybrid organelles formed by MVE and lysosomes [35–37]. Lysosomes are localized predominantly in the juxtanuclear region, where receptor-containing endosomes are transported along microtubules [38, 39]. Thus, the EEA1-mediated dynamics of early endosomes homotypic fusions, PI3P-dependent formation of MVE, translocation in the juxtanuclear region and delivery of EGF-EGFR to lysosomes can be considered as hallmarks of the degradative pathway affording a convenient comparison of the behavior of different ligand-receptor complexes.

Note that the main concepts on the dynamics of EGFR endocytosis have been formed substantially on the basis of immunofluorescence approaches by protocols including cell fixation at different time points after endocytosis stimulation [21, 23]. However, a certain fixation technique can significantly affect the state of membranes or polymers like cytoskeleton. Obviously, the use of labels that allow to avoid fixation is very attractive, thus QDs with their photostability and brightness may be a perfect tool to detect intracellular distribution of the labeled ligand-receptor complexes during endocytosis. Additionally, QD's properties make it possible to carry out long-lasting experiments on live cells. A comparison of the data obtained simultaneously on fixed and live cells is extremely important to understand how current ideas about the mechanisms of endocytosis regulation correlate with the processes in live cells, however, such studies are practically absent. Also there is no systematic studies of EGF-QD endocytosis pathway in comparison to that of native EGF made by standart confocal microsopy analysis of fixed cells.

In our previous work we have found that biotin-EGF complexed to streptavidin-QDs (bEGF-savQD) compared to native EGF and bEGF causes insignificant differences only at the stage of binding to EGF receptors. We have shown that most “endocytically efficient” bioconjugates are formed at EGF:QD concentrations ratio of 6–4:1 [40]. So, the found differences are most probably due to spatial problems of formation of biologically relevant receptor dimers of the QD-EGF-EGFR complexes on the cell surface. However, once formed, such ligand-receptor complexes are internalized via EGF-, EGFR- and clathrin-dependent way characteristic for native EGF. Also, QD implementation did not change the dynamics of EEA1 recruitment to endosomes and their interaction with HRS, the first ESCRT0 component indicative for entry into lysosomal degradative pathway [40]. Thus, we have concluded that QDs do not significantly affect the very early stages of endocytosis. However, taking into account that the half-life of EGF-receptor complexes on the surface is at least an order of magnitude less than upon their internalization up to delivery to lysosomes, a further detailed study seems to be very important to conclude about possible side effects at the late stages of endocytosis. Obviously, introduction of QDs in research practice requires a better understanding of the possible limitations of their use, both in basic research and in applied fields.

According to the above-mentioned, the main aim of the present work was to compare the dynamics of endocytic events, stimulated by the native EGF and by bEGF-savQD after internalization during the early and late stages of endocytosis, including (i) early endosome fusions, (ii) PI3P-dependent endosomal maturation into MVE, (iii) endosome translocation to the juxtanuclear region and (iv) interaction with lysosomes as the key ones. At first, the dynamics of EEA1 association with endosomes loaded by the native and QD-bound ligand was evaluated as a marker of fusion activity, necessary for the following MVE formation. Maturation process was studied in pulse-chase-pulse experiments to evaluate the fusion ability of the endosomes formed during two pulses with increased intervals between them. MVE formation was probed by the inhibitory approach using wortmannin, inhibitor of Vps34. We also analyzed the dynamics of endosome translocations both on fixed cells (using the perinuclear index dynamics as a measure of resultant translocations) and on live cells (by visualization of endosome motions in real time). Finally, the course of ligand-receptor complexes delivery to lysosomes and receptor degradation were analyzed for the both ligands. The data obtained suggest that the use of QDs enables adequate description of the labeled molecules’ behavior, when they enter the cell via endocytosis. Additionally, QDs are very useful for studying of such highly dynamic processes as transport of QD-labeled vesicles in real time.

## RESULTS

### The dynamics of endocytosis stimulated by native EGF or bEGF-savQDs complexes

**Figure 1:**
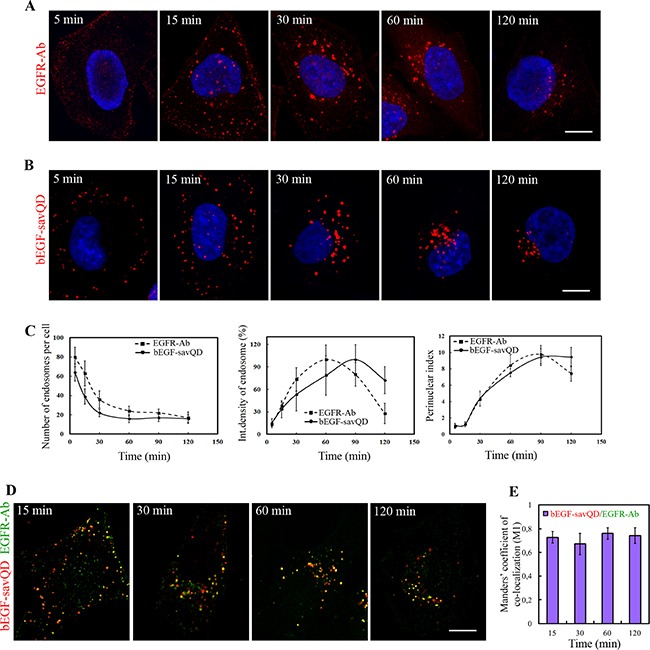
Time-dependent imaging of EGFR and EGF-QDs distribution in HeLa cells HeLa cells were incubated with (**A**) 2 nM EGF or (**B**) bEGF-savQD (prepared by incubation of 2 nM bEGF with 0.5 nM QDs) using pulse-chase protocol for the indicated time. (A) At each time point, the cells were fixed and immunostained with anti-EGFR antibody (Alexa 568) and DAPI before confocal microscopy. (B) For each time point a new well from the chambered cover glasses were used and images were taken in live cells without fixation. The nuclei were stained with Hoechst 33258. (**C**) The number of endosomes, their integrated densities and perinuclear index per cell (from the experiment described in A and B) were calculated for each time point using ImageJ. (**D**) HeLa cells were incubated with bEGF-savQD (2:0.5 nM) using pulse-chase protocol for the indicated time. Then the cells were fixed and immunostained with the anti-EGFR antibody (Alexa 488) before confocal microscopy. Co-localizations between bEGF-savQD and EGFR-Ab were quantified using Manders’ coefficient (M1). Each image is representative for the corresponding time point of at least three independent experiments. The number of endosomes, their integrated densities and perinuclear index per cell are presented as the mean ± 95% confidence interval. Scale bars: 10 μm.

As mentioned earlier, the bulk of widely recognized data on the dynamics of EGFR endocytosis was obtained by a canonical approach that includes endocytosis stimulation by ligand addition followed by the cells’ fixation at several time points and staining them with a receptor-specific antibody. Importantly, application of this protocol for the analysis of endocytosis stimulated by bEGF-savQD does not require any fixation procedure that allows to avoid possible distortions and to detect labeled EGF-EGFR complexes in live cells under conditions that do not disturb the organization of main organelles and structures like cytoskeleton. To test whether bEGF-savQD endocytosis differs in any way from endocytosis of EGFR in HeLa cells, we followed simultaneously the course of endocytosis of EGF-receptor complexes, detected by staining with Abs upon fixation, and of bEGF-savQD (by direct detection of QD fluorescence) using confocal microscopy imaging (Figure 1A and 1B). In both cases EGFR (Figure 1A) or QDs (Figure 1B) were observed intracellularly in the close proximity to the plasma membrane soon after the addition of the corresponding ligand to the cells. After 15 min endosomes containing EGFR or QDs were distributed evenly throughout the cytoplasm, and most of them were still located near the plasma membrane. After 30 minutes, enlarged endosomes were detected in the perinuclear region in the both cases. After 60–120 min, almost all endosomes concentrated in the juxtanuclear region.

The dynamic of the overall process was quantitatively estimated by the image analysis for the both cases using parameters which characterize the number of endosomes, their mean intensities and “perinuclear index” (Figure 1C). While the first two values reflect the processes of fusions and cargo concentration, the third parameter, calculated as the ratio of the total cell area to the area where most of endosomes are localized in the cell, is the measure of endosome transport efficacy toward the microtubule-organizing center. It can be seen that in both cases the number of endosomes drops significantly up to 60 min of endocytosis stimulation, while mean integrated density of an endosome increases, which reflects a similar fusion dynamics without any statistically significant difference. In both cases the mean integrated density of the label passed through the maximum, which is slightly delayed for bEGF-savQD. Importantly, at 120 min the mean integrated density of EGFR-containing endosomes has fallen to about 28% of the maximum, which is interpreted as EGFR degradation in lysosomes [21]. However, the mean integrated density of bEGF-savQD-containing endosomes decreased only slightly and was about 72% of the maximal value (Figure 1C). There could be several explanations for this observation: from dysfunctions in endosomal maturation or endosome-lysosome interactions to QD stability in lysosomes, therefore we have further focused on these issues in detail.

The dynamics of endosomes’ accumulation in juxtranuclear region estimated by growth of perinuclear index during endocytosis demonstrated no significant difference for receptor complexes formed by the both ligands: most intensively translocations occurred between 15 and 60 min after endocytosis stimulation (Figure 1C).

Notably, when endocytosis was stimulated by EGF a typically slight diffuse surface staining could be detected after 5 min pulse (Figure 1A) that was absent in the case of bEGF-savQD (Figure 1B). Such staining demonstrates that the antibody technique results in the detection of the total receptor population while QDs label only the receptors complexed to the ligand. Indeed, bEGF-savQD stays in complex with EGFR long after internalization in endosomes, which is proved by the high value of Manders’ co-localization coefficient (M1): practically all QD-positive structures contain the EGFR signal during 120 min after endocytosis stimulation (Figure 1D). This proves that at each analyzed time points QDs, EGF and the receptor are localized predominantly in the same vesicular structures in the cell. This fact makes possible a comparison of the data obtained on live cells using bEGF-savQDs complexes with the data obtained on fixed cells by means of EGFR antibodies, which is in agreement with our earlier observations [40].

Dynamics of endocytosis was also studied on epithelial carcinoma cell line A549 (Supplementary Figure 1). These cells demonstrate the same typical features of EGF-EGFR endocytosis: the decrease in endosome number per cell at early stages correlates with the increase in endosomal apparent size and brightness. The latter reaches its maximum by 30 min and then decreases. Also, accumulation of endsomes in juxtanuclear region is registered. It is necessary to note, that the exact dynamics was slightly different from that in HeLa cell: EGFR was detected in these cells even at 150 min after endocytosis stimulation while in HeLa cells EGFR was practically degraded by 120 min (compare Figure 1A, 1C and Supplementary Figure 1A, 1C). However, up to these times in the both cell lines QDs demonstrated extremely high level of co-localization with EGFR (Figure 1D, 1F and Supplementary Figure 1D, 1F). Most important point is that in spite of some differences in endocytosis dynamics the behavior of the two tested ligands, EGF and bEGF-savQD, in the both cell lines were practically the same.

### Endosomes containing native EGF or bEGF-savQD undergo fusions in a similar way

**Figure 2:**
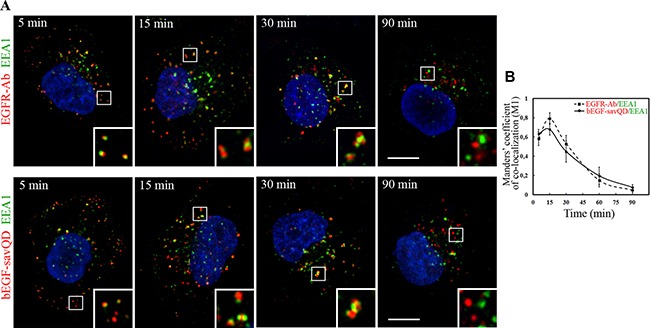
Immuno-co-localization of EGFR and bEGF-savQD with EEA1 (**A**) HeLa cells were incubated with 2 nM EGF or bEGF-savQD (2:0.5 nM) using pulse-chase protocol for the indicated time. Then cells were fixed and immunostained with the anti-EGFR (Alexa 568) and anti-EEA1 (Alexa 488) antibodies or in the case of bEGF-savQD with anti-EEA1 (Alexa 488) antibody before confocal microscopy. The nuclei were stained with DAPI. The insets represent enlarged views (3×) of the corresponding boxed region of the cell. Each image is representative for the corresponding time point of at least three independent experiments. Scale bars: 10 μm. (**B**) Co-localizations between EGFR-Ab or bEGF-savQD and EEA1 were quantified using Manders’ coefficient (M1). The data presented as the mean ± 95% confidence interval of three independent experiments.

The dimeric protein EEA1 that provides tethering of homotypic early endosomes at the first stage of fusion is widely used as the early endosome marker [41, 42]. The protein is thought to be recruited to the receptor-containing early endosomes after endocytosis stimulation and associated with them until they mature into the late ones. As shown above, the initial number of vesicular structures containing bEGF-savQD per cell decreased 3-fold by 30 min of incubation, and their integrated density increased 6-fold, indicating efficient endosome fusions (Figure 1C). We analyzed the dynamics of association of vesicles containing bEGF-savQDs or EGFR with EEA1 in HeLa (Figure 2) and A549 (Supplementary Figure 2) cells which was found to be quite similar for the two cell lines. Indeed, the both ligands in the two cell lines were detected in small vesicular endosomes with EGFR or bEGF-savQD containing EEA1 several minutes after endocytosis stimulation. Later on, the endosomes in which EGFR/QD and EEA1 domains are clearly seen become larger and gain an irregular shape other than round. After 90 min of stimulation the endosomes containing only EGFR/bEGF-savQDs or only EEA1 are observed, i.e. EEA1 and EGFR/bEGF-savQD are localized on different structures in both cell lines (Figure 2A and Supplementary Figure 2A). These data are also confirmed by the dynamics of Manders’ co-localization coefficient (M1), which reaches the maximum at 15 min and decreases after 30 min in the case of the EGF-, as well as of bEGF-savQD-stimulated endocytosis (Figure 2B and Supplementary Figure 2B). Importantly, these different types of structures with separated EEA1- and ligand-enriched domains as well as their shape changes have the same appearance and dynamics for both ligands. These results suggest that QD-labeled EGF does not affect fusogenic activity and maturation of endosomes.

**Figure 3:**
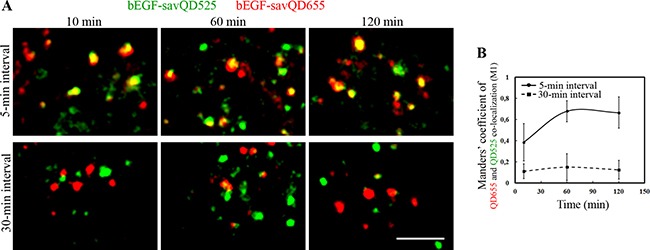
Analysis of endosome fusions in HeLa cells (**A**) bEGF-savQDs with different emitting wavelengths 525 and 655 nm were added sequentially. Initially, bEGF-savQD525 were added for 5 min at 37°C, then the cells were washed and chased for an additional 5- or 30-min intervals; then bEGF-savQD655 were added for 5 min at 37°C, the cells were washed and chased for the indicated time periods. For each time point a new well from the chambered cover glasses were used and images were taken in live cells without fixation. Each image is representative for the corresponding time point of at least three independent experiments. Each image in a panel presents a part of a cell typical for indicated time point. Scale bars: 10 μm. (**B**) Co-localizations between bEGF-savQD655 and bEGF-savQD525 for 5-min and 30-min intervals were quantified using Manders’ coefficient (M1). The data presented as the mean ± 95% confidence interval of three independent experiments.

Another established test is based on the loss of similarity of endosomal membrane composition with time due to maturation, which results in the inability to fuse with the just formed endosomes. To check if this is true for bEGF-savQDs we have used a pulse-chase-pulse experiment, when first bEGF-savQD525 with green fluorescence and then bEGF-savQD655 with red one were added to the cells for 5 min with different chase intervals between two pulses and co-localization due to fusions was estimated. When the interval between pulses was 5 min, the portion of vesicles containing the both green and red signals was already observed 10 min after the second pulse (Manders’ co-localization coefficient (M1) is 0.38), and the number of yellow endosomes grew during the next 60 min (M1 co-localization coefficient reaches 0.68) (Figure 3). On the contrary, at 30-min interval co-localization between green and red endosomes was almost absent during all 120 min of endocytosis (Figure 3A). The Manders’ coefficient (M1) in this case was very low, and it did not changed at the later stages of incubation (Figure 3B), thus indicating the absence of endosome fusions. Thus, the data obtained in live cells confirmed the previously reported criterion on permitted fusions only between endosomes at a similar stage of maturation [43]. Our results also show the advantage of such approach to study fusions in cases when vesicles are too small to estimate their interactions on the basis of size changes: indeed, the just formed endosomes are about 100–200 nm in diameter, which is less than the resolution limit of light microscopy. However, one should take into account that this protocol underestimates the number of fusions, excluding those happens between endosomes carring QDs of the same color.

### Endosomes containing bEGF-savQDs normally form multivesicular endosomes

Formation of multivesicular, or late, endosomes (MVE), is one of the main late stages of endocytosis, associated with endosome maturation. MVE originate from previously enlarged endosomes by forming invaginations in the membrane regions enriched in PI3P, which then turn into vesicles budding inside the endosome. This process should evidently decrease the endosome's size, but this decrease for such small objects as endosomes can not be exactly revealed by optical microscopy. Thus, we did not estimate these apparent sizes but focused on mean integral densities of endosomes, the parameter indicative for cargo concentrating. In our experiments on HeLa cells mean apparent size of vesicles grew only up to 30 min, but their mean integrated density reached its maximum at 60–90 min (compare A-B panels and corresponding plot in C panel of Figure 1). These data are in agreement with cargo concentration due to MVE formation.

**Figure 4:**
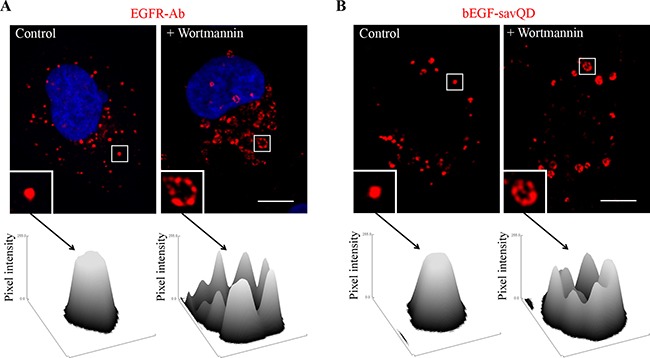
Effect of wortmannin on EGFR endocytosis stimulated by EGF or bEGF-savQDs Control or wortmannin-treated (100 nM) HeLa cells were incubated with (**A**) 2 nM EGF or (**B**) bEGF-savQD (2:0.5 nM) using a pulse-chase protocol for 60 min. Then, for (A), the cells were fixed and immunostained with the anti-EGFR antibody (Alexa 568) and DAPI before confocal microscopy. For (B), live cells were analyzed and for each case a new well from the chambered cover glasses were used. The insets represent enlarged views (2.8 ×) of the corresponding boxed region of the cell. Each image is representative for the corresponding time point of at least three independent experiments. Scale bars: 10 μm. For each indicated endosome the surface plot of the intensities of pixels was plotted using ImageJ.

However, wortmannin will prevent invaginating by inhibiting PI-3-kinase Vps34, one of the key regulators of MVE internal vesicles formation [44]. Thus, the inhibition should result in the formation of enormously enlarged hollow vesicles. Indeed, we demonstrated that such structures were revealed in the presence of wortmannin 60 min after endocytosis stimulation by the native EGF, as well as in the case of bEGF-savQDs (Figure 4). It is clearly seen that unlike controls controls, after wotmannin treatment EGFR as well as bEGF-savQD are not found inside the vesicles, being located only on the outer membrane of endosomes (Figure 4, surface plots). Interestingly, that inability to form MVE does not affect endosome motility as the enlarged immatured structures are located in the same juxtanuclear regions as normally formed MVE in control cells (compare Figure 4A, 4B and Figure 1A, 1B). Note that the processes of MVE formation and its inhibition by wortmannin detected in live cells (bEGF-savQD) and upon fixation (EGFR-Abs) were similar.

### Visualization of bEGF-savQD-containing endosomes motility. Live cell imaging

**Figure 5:**
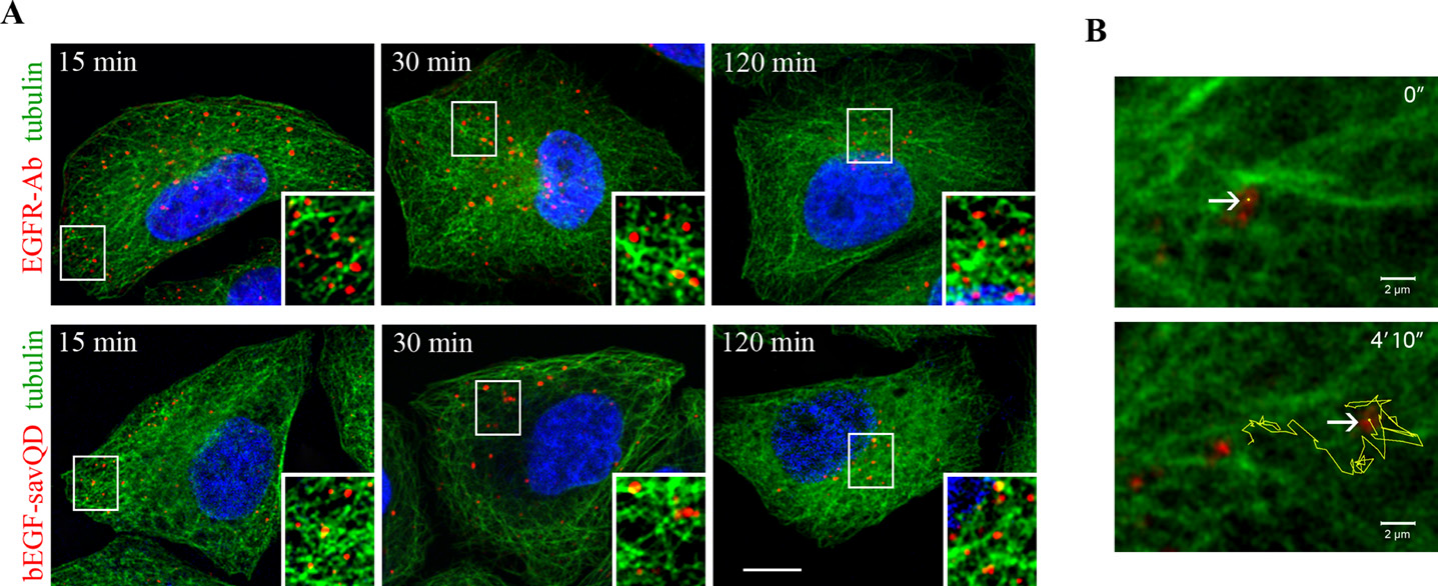
Distribution of EGFR and bEGF-savQDs in relation to the microtubules HeLa cells were incubated with (**A**) 2 nM EGF or bEGF-savQD (2:0.5 nM) using a pulse-chase protocol for the indicated time. Then the cells were fixed and immunostained with anti-EGFR (Alexa 568) and anti-α-tubulin (Alexa 488) antibodies or in the case of bEGF-savQD cells were stained with anti-α-tubulin (Alexa 488) antibody before confocal microscopy. The nuclei were stained with DAPI. The insets represent enlarged views (2.1 ×) of the corresponding boxed region of the cell. Each image is representative for the corresponding time point of at least three independent experiments. Scale bars: 10 μm. (**B**) The first and last frames from a representative time-lapse video (see Supplementary Video 1) illustrating the motility of the endosome bearing bEGF-savQDs in relation to the microtubules show the trajectory of endosome movement 30 min after endocytosis simulation by bEGF-savQD in HeLa cells expressing GFP-α-tubulin.

It is well established that endosomes move from the cell periphery to the juxtanuclear region along the microtubules by means of minus-end motor dynein [45–47]. Indeed, when HeLa or A549 cells were stimulated to endocytose EGF and then fixed at different time points, re-localization of endosomes toward the nucleus and accumulation in juxtanuclear area were clearly seen (Figure 1A, 1B and Supplementary Figure 1). Importantly, the overall dynamics of this accumulation, estimated by normalized value of decrease of area occupied with vesicles (perinuclear index), was the same in the case of bEGF-savQDs, when confocal images were taken at the same time points in live cells without fixation (Figure 1A-1C). When the cells stimulated by EGF or bEGF-savQDs and fixed at the indicated time points were stained also with anti-tubulin antibody, endosomes were found on the microtubules or in their close proximity during all 120 min of endocytosis (Figure 5A). So, endosomes containing bEGF-savQDs demonstrate exactly the same behavior as EGFR-containing endosomes, and on basis of fixed cells analysis it can be described as uniform linear movement.

However, when the motility of endosomes bearing bEGF-savQDs was studied in real-time in live HeLa cells expressing GFP-α-tubulin at 37°C (Figure 5B, Supplementary Video 1) quite a different pattern of endosome movement was found. Relatively short linear translocations of selected single endosome directed both “forward” and “backward” but resulted in a long “forward” translocation for about 7 μm have been registered. Then the endosome lost the track (at about 1’50” from the beginning of registration) and started to move chaotically for very short distances inside restricted area. During the remaining time the endosome moved in two such areas jumping from one to the other by 2’30’’. As a result, during about 4 min of registration the endosome was translocated for about 7 μm straight from initial to final point, but physically it has covered a several fold longer way. As for about other endosomes followed in the cells, the straight distances may be shorter or longer, but in general the character of movements was the same and looked somewhat chaotic with periods of fast directed runs taking a relatively low part of its lifetime (data not shown). Such combination of chaotic fluctuations with relatively long linear runs is typical for microtubule-facilitated motion and usually is described as repeated periods of diffusive and active traffic [48].

Notably, during the registration period it were GFP-labeled microtubules but not QDs that significantly lost in brightness due to photobleaching. Also it is important to stress that microtubule network seen in video is rather dense and very flexible: the initial “landscape” significantly differs from final one. We can conclude that QD-labeling of vesicles provide useful tool for studies that will further help to uncover the real dynamics of the translocation process.

### QDs complexed to EGF cause a delay in EGFR degradation at the late stages of endocytosis

**Figure 6:**
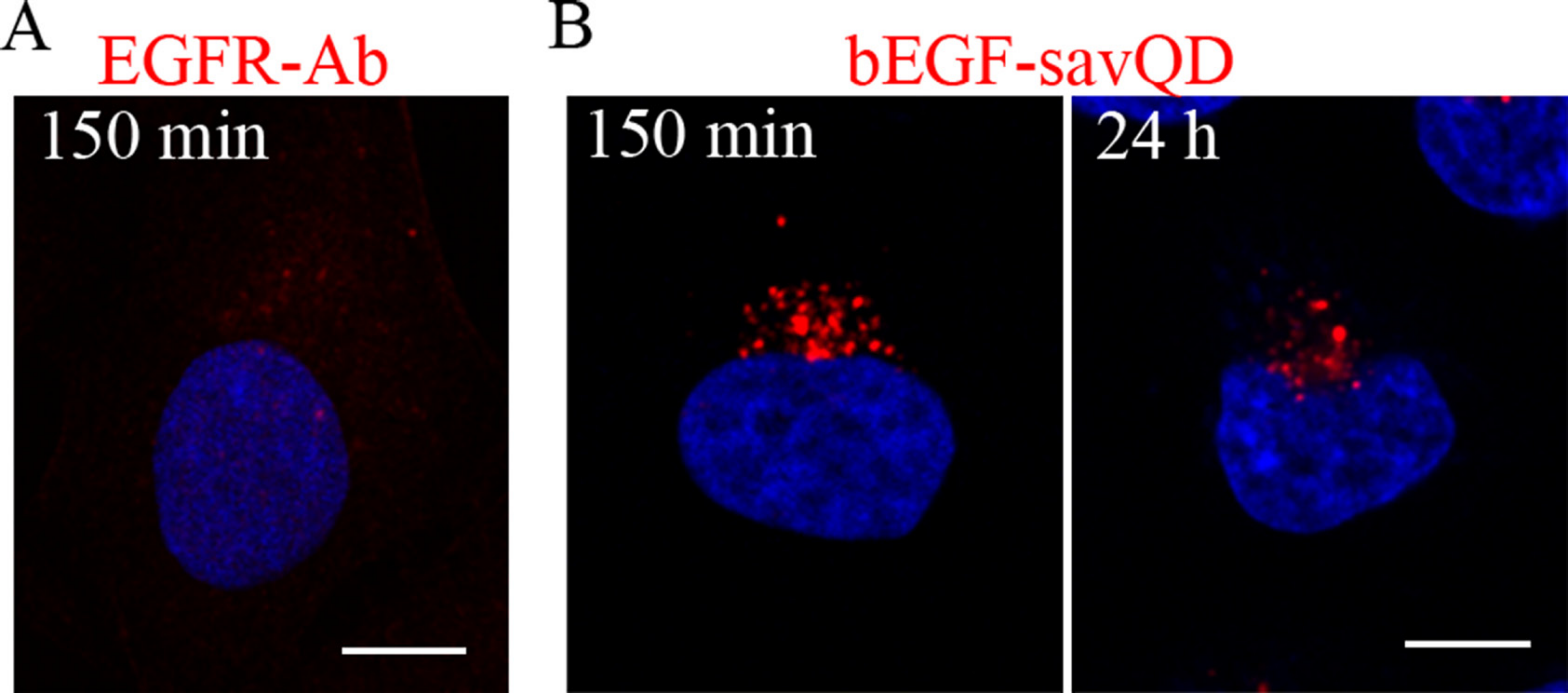
QDs entering HeLa cells by EGFR-mediated endocytosis are revealed intracellularly for 24 h HeLa cells were incubated with (**A**) 2 nM EGF or (**B**) bEGF-savQD (2:0.5 nM) using a pulse-chase protocol for the indicated time. Then in (A) the cells were fixed and immunostained with anti-EGFR antibody (Alexa 568) and DAPI before confocal microscopy. In the case (B) of bEGF-savQD stimulation live cells were analyzed from a new well from the chambered cover glasses for each time point. The nuclei were stained with Hoechst 33258. Each image is representative for the corresponding time point of at least three independent experiments. Scale bars: 10 μm.

It is well known that the late multivesicular endosomes interact with lysosomes and both EGF and its receptors are degraded [21, 36, 49]. This usually occurs 90–120 min after endocytosis stimulation. Indeed, in our experiments in HeLa cells at 150 min after stimulation by EGF only a few small dim receptor-containing structures were revealed by the receptor-specific antibody, indicating degradation of the major portion of internalized EGFR in lysosomes by this time (Figure 6A). In contrast, when endocytosis was stimulated by bEGF-savQDs, rather large and bright juxtanuclear structures containing QDs were detected in the cells after 150 minutes. Moreover, they were still detected in the cells even after 24 h, although their number and intensity decreased (Figure 6B). In A549 cells more receptor-positive structures could be detected at 150 min, but no EGFR was revealed at 24 h, despite the presence of bright QD-bearing structures (Supplementary Figure 1).

Obviously, a semiconductor particle can not be destroyed by lysosomal hydrolases, but the revealed differences raise a question whether bEGF-savQDs do reach lysosomes or whether QD complexed to EGF and its receptor in lysosomes interferes with the availability of the protein moiety to hydrolases.

**Figure 7:**
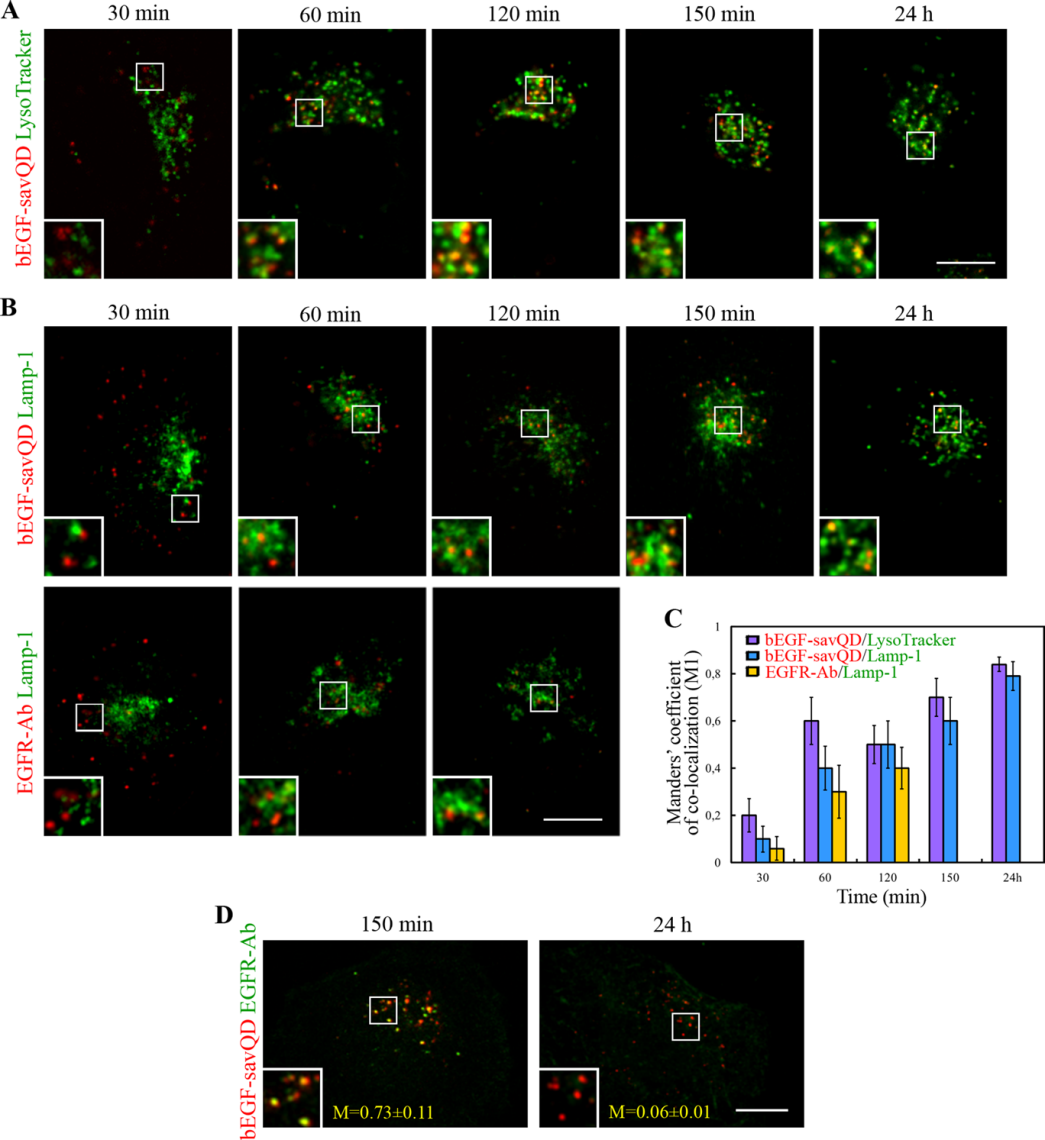
Analysis of interaction of the endosomes containing bEGF-savQDs or EGFR with lysosomes HeLa cells were incubated with bEGF-savQD (2:0.5 nM) or 2 nM EGF using a pulse-chase protocol for the indicated time. (**A**) Live cells were analyzed and for each time point a new well from the chambered cover glasses were used. LysoTracker Green DND-26 was added into the culture medium for 20 min prior to confocal imaging. (**B**) At each time point the cells were fixed and immunostained with Lamp-1 (Alexa 488) or with anti-EGFR (Alexa 568) and Lamp-1 (Alexa 488) antibodies before confocal microscopy. The insets represent enlarged views (2.3 ×) of the corresponding boxed region of the cell. (**C**) Co-localizations between bEGF-savQD or EGFR-Ab and lysosomal markers were quantified using Manders’ coefficient (M1). The data presented as the mean ± 95% confidence interval of three independent experiments. (**D**) HeLa cells were incubated with bEGF-savQD (2:0.5 nM) using a pulse-chase protocol for 150 min and 24 h. Then the cells were fixed and immunostained with anti-EGFR (Alexa 488) antibody before confocal microscopy. Manders’ coefficients (M1) are designated. The insets represent enlarged views (2.3 ×) of the corresponding boxed region of the cell. Each image is representative for the corresponding time point of at least three independent experiments. Scale bars: 10 μm.

To shed light on this problem, a simultaneous detection of bEGF-savQDs and fluorescent probe LysoTracker allowing to trace lysosomes in live cells was carried out (Figure 7A). From 60 min, a high degree of co-localization of QD-containing structures with LysoTracker was observed (Figure 7C). However, some co-localization was detected at the early stages of endocytosis. This could be explained by LysoTracker accumulation in all acidified organelles including late endosomes. So, we additionally analyzed the same endocytosis stages using a more specific lysosomal marker Lamp-1 in the cells stimulated by QD-labeled EGF as well as by the native EGF (Figure 7B). At 30 min after endocytosis stimulation a low co-localization of QD-containing vesicles with Lamp-1 was revealed but it increased with incubation time as with LysoTracker (Figure 7B and 7C). Notably, in the case of endocytosis stimulation by EGF, the dynamics of co-localization of EGFR with Lamp-1 from 30 to 120 min was similar to that of bEGF-savQDs (Figure 7B and 7C).

This means that EGF complexed to QD is delivered into lysosomes with the same dynamics as the native EGF. However, as it was shown, after 120 min the internalized EGFR was completely degraded (Figure 6A) whereas the QD-containing structures were accumulated in lysosomes and even after 24 h QD signals were still co-localized with LysoTracker and Lamp-1 (Figure 7A and 7B). Moreover, Manders’ co-localization coefficients (M1) became 60 % higher compared to that at 120 min (Figure 7C). Importantly, the staining of the cells internalized bEGF-savQDs with the EGFR-specific antibody demonstrated that at 150 min QD-positive vesicles contained significant amount of EGFR (mean value of M1 is 0.73), but by 24 h these structures became receptor-negative (Figure 7D). This means that EGFR is eventually degraded, but its degradation is delayed compared to the cells with the native EGF-EGFR complexes.

Comparison of co-localization of tested ligands in two cell lines, HeLa and A549, show different dynamics of late endocytic stages (Supplementary Figure 3). Indeed, while in HeLa cells Manders’ coefficient grew constantly from 0.2 to 0.7 up to 120 min (Figure 7C), in A549 it reached a plateau of about 0.4 at 30 min and then did not change up to 150 min (Supplementary Figure 3C). These data correlate with longer preservation of EGFR protein in A549 cells, which can be due to certain features of endosome-lysosome interactions in these cells. However, EGF and bEGF-savQD demonstrate the same differences in behavior that were described for HeLa cells.

## DISCUSSION

Earlier we have shown that bEGF-savQD specifically interacts with the EGF receptors and enters HeLa and A431 cells via receptor-mediated clathrin-dependent endocytosis, typical for the native EGF [40]. Here we have compared the intracellular fate of internalized ligand-receptor complexes formed by the native EGF with that formed by bEGF-savQD. Since EGF receptor activity is known to play a key role in the regulation of both the endocytic pathway and receptor signaling, we assumed that endocytosis dynamics similar to the canonical one may be an indirect marker of the proper signaling. Here, we have demonstrated that EGFR is revealed in endosomes loaded with both types of ligand up to its delivery to lysosomes.

For a more detailed comparison we have chosen several critical events on the endocytic pathway known to be governed by the receptor tyrosine kinase activity: (i) EEA1-mediated fusions of early endosomes as a prelude of maturation process, (ii) endosomal maturation *per se* indicated by PI3P-dependent formation of MVEs and the loss of fusion ability between heterotypic endosomes, (iii) microtubule-facilitated translocation in the juxtanuclear region where the majority of lysosomes are localized and (iv) delivery to lysosomes.

We have demonstrated that in comparison with the native EGF, QD-conjugated EGF promoted the same dynamics of association and, importantly, dissociation with the tether protein EEA1 involved in the first step of the fusion process (Figure 2 and Supplementary Figure 2). This means that the early stage of endosomal processing is similar for the both ligands. Moreover, endosomes containing bEGF-savQDs were able to fuse at the early stages of endocytosis if the two pulses of ligands were added shortly one after the other but they lost this ability as the interval between the additions of the ligands increased (Figure 3). When the chase time was 5 min, the co-localization of “green” and “red” QDs was high, but when this interval was increased up to 30 min, co-localization was very low indicating that during this time the membranes of QD-containing vesicles undergo significant changes, or mature, moving along the endocytic pathway, and are no longer able to fuse with the newly formed vesicles (Figure 3). These data are entirely consistent with the view that the early stage of endosome maturation is connected with their fusions, thus allowing to increase the surface area and then to form multivesicular structures. During this time, the early markers leave endosomes by recycling back to the plasma membrane and the endosomal membrane changes its properties acquiring the newly synthesized late markers from the trans-Golgi network. Our data are fully consistent with the maturation model of Murphy [43] which argues that the early endosomes are gradually transformed into the late endosomes and lysosomes.

Importantly, during the early fusions the endosome size is about 100–200 nm, which is under the resolution limit of conventional light microscopy and it is impossible to detect a fusion of any two vesicles based on their visible size changes. However, these fusions can be reliably demonstrated using one of the advantages provided by QDs: a small change in the particle core size results in a significant difference in the emission wavelength. Since the final size of a QD (15–20 nm) is determined mostly by functionalizing layers of PEG and streptavidins, the increase in CdSe/ZnS core size for 2–4 nanometers has a negligible input, but it is enough to change the emission light from green (525 nm) to red (665 nm). So, the addition of bEGF-savQD525 followed by bEGF-savQD665 allowed estimating fusions by the appearance of the yellow color thus indicating co-localization of the two labels (Figure 3). This approach also works when small vesicles fuse with a larger one.

We have also shown that an increase in the size of the bEGF-savQD-EGFR complex compared to that formed by the native EGF does not affect the process of invaginations and pinching off of the internal vesicles leading to the formation of MVEs (Figure 4). This result was expected because during the invagination process the extracellular portion of the ligand-receptor complex is oriented toward the lumen of MVE, but not in the lumen of a small internal vesicle, thus the enlargement of the ligand by QD implementation should be neutral. According to the manufacturer's statement savQD is about 15–20 nm in diameter [50]. Importantly, in the recent paper of [51] it was shown that EGF-complexed nanoparticles resulted in a sufficient delay of endosome maturation and consequent increase in the caspase activity. Basing on the above-mentioned, the authors suggest nanoparticle involvement in the apoptosis stimulation. However, they used nanoparticle of 80 nm in diameter that should obviously interfere with the formation of the internal vesicles of MVE, which are usually about 50 nm [52–54]. Indeed, in this case it is quite expectable that TK activity of the endosomal EGF receptor complexes will last abnormally. These results once again underline the great significance of the signal duration and indicate that the nanoparticle size is very important for the correct maturation process.

In our recent paper [40] we have demonstrated that bEGF-savQDs form endosomes that acquire HRS, a component of the ESCRT0 sorting complex, with a dynamics similar to that of the native EGF that manifests entering the degradative pathway. Taken together, our data show that the maturation process of endosomes containing bEGF-savQDs does not differ from the maturation of endosomes in the case of endocytosis stimulation by the native EGF. Thus, an important conclusion can be made that during endocytosis bEGF-savQDs behave as a neutral marker for the degradative pathway and can be used safely.

This is also true for the studies of endosome translocation. Moreover, QD photostability provides an additional perfect opportunity to follow the real processes in real time. A canonical way to prove endosomal trafficking was to register vesicle distribution at several time points after endocytosis stimulation by fixing the cells and staining them for endosomal or cargo markers and microtubules as presented in Figures 1 and 5A. However, this approach shows a common trend and just a summary of the process, while the real behavior of transported endosomes can be studied only by using live cell imaging (Figure 5B and Supplementary Video 1). We see that the mobility pattern of QD-labeled endosomes is much more complicated compared to the model of the uniform unidirectional process, which could only result from the canonically obtained data. In general, the cycles of fast directed runs followed by oscillations inside restricted areas, are characteristic for microtubule-dependent transport of endosomes, aggresomes and secretory granules and was described elsewhere [47, 48, 55]. The reasons for such behavior are still under debate, but a detailed discussion of these issues is beyond the scope of the present paper. In brief, the “tug-of-war” concept [56] that is based on motor protein competition (the both kinesins and dynein are found on endosomes), is the most popular one, but there may also be some other explanations. Note how flexible and confused the microtubule network is (Supplementary Video 1), so this network could be both rails to ride along and an obstacle for the direct movement for long distances. Additionally, these complicated motility patterns can explain why translocation of endosomes into the juxtanuclear region usually takes about 30–90 min instead of several minutes taking into account the high speed of dynein-mediated transport [47].

So, finally endosomes are delivered to the neighborhood of the microtubule-organizing center where, according to the existing view, they interact with lysosomes. Indeed, 60–120 min after endocytosis stimulation, endosomes with bEGF-savQDs and endosomes with EGF receptor were found there. The analysis of co-localization of bEGF-savQD- and EGFR-containing structures with Lamp-1 at 120 min revealed Manders’ coefficients (M1) close to 0.5 (Figure 7C). However, these similar values may have a different rationale and thus be interpreted in a different way. In the case of endocytosis stimulation with the native EGF, the fusion of the late endosomes with lysosomes leads to a rapid degradation of the receptors [21, 36, 49] and, therefore, to a rather limited in space and time co-localization of the receptor and the lysosomal marker. Thus, the real amount of EGFR delivered to lysosomes could be much higher than the M1 value of about 0.5 reflects. Indeed, at 150 min no EGFR is detected in Lamp1-positive structures showing a complete degradation of the protein. However, in the case of bEGF-savQDs, as we have mentioned above, fast disappearance of the semiconductor particle due to degradation could hardly be expected and the M1 value of about 0.5 may be interpreted as a delayed delivery of the complexes to lysosomes compared to the native EGF. An increase in Manders’ coefficient (M1) for bEGF-savQD and Lamp-1 up to the maximum value of 0.8 at 24 h of incubation (Figure 7B and 7C) supports this idea. Additionally, we have demonstrated that EGFR bound to bEGF-savQD is not degraded at the very late stages of endocytosis for a longer time than EGFR bound to the native EGF in the both cell lines tested (Figure 7 and Supplementary Figure 3). Altogether, these data suggest that bEGF-savQD-EGFR delivery to lysosomes and degradation of the proteinous portion of the complex are delayed by QD implementation, but not inhibited. These observations could be of great importance, given that the late endosomes are associated with the second wave of activation of MAP kinases ERK1/2, Akt-dependent activation of anti-apoptotic cascade, and stimulation of P21^waf^ expression [57–61]. If the reported here delay takes place at this stage, late signaling will increases its duration, which should disturb the physiological response. However, one should take into account that inclusion of bEGF-savQDs into the internal vesicles of MVE has quite a similar dynamics with the native EGF (Figure 4). This means that at the late stages (90 min and later) practically all EGFR, localized initially onto the outer endosomal membrane and thus signaling-competent, is translocated inside an endosome and becomes isolated from cytoplasm. Thus, differences in the dynamics of the late stages should be of no importance for EGFR signaling outcome, but the reasons for these effects should be undoubtedly studied further in greater detail. We do not know of any studies addressing this aspect of bEGF-savQD signaling. Though some studies have been published where the intracellular processes were probed using QD-labeled ligand, and the authors suggested *a priori* that the behavior of the native and QD-conjugated one were identical [11, 15]. Indeed, these groups successfully applied QD-labeled ligands for the analysis of endosomal mobility.

An expected difference was uncovered by demonstrating that savQDs conjugated with transferrin (Tf) were internalized normally by clathrin-dependent endocytosis as fast as the native Tf, but savQD-bTf-receptor complexes recycling was blocked [12]. This is most probably due to spatial problems of large savQD-bTf moiety packing into narrow recycling tubules. EGFR is known to be able to recycle, and the degree of recycling is proportional to the expression level of the receptor [22, 24]. These recycling receptors, in turn, maintain mitogenic signaling from plasma membrane. Considering the fact that many gastrointestinal, lung and other epithelial malignancies are associated with the overexpression of the EGF receptor, the use of bEGF-savQDs in low concentrations reveals the possibility of identifying the transformed cells without triggering additional proliferation usually caused by recycling. Moreover, as bEGF-savQDs are efficiently concentrated in lysosomes, detection of such cells at the earliest tumor stages, at the level of individual cells, become possible. Also, the large size of QDs provides enough space to add photosensitizer to destroy cancer cells specifically.

Thus, our data suggest that bEGF-savQDs can be a reliable marker for basic studies of endosomal formation and all aspects of ligand-receptor complexes progression along the degradative pathway, but these complexes should be used with caution when addressing recycling or lysosomal degradation process itself. However, these problems can turn back the benefits in case of practical use for early diagnostics of some types of cancer or photodynamic therapy and other applications.

## MATERIALS AND METHODS

### Reagents and antibodies

Epidermal growth factor biotin conjugate (bEGF), CdSe/ZnS Qdot streptavidin conjugate emitted at 655 nm and 525 nm (savQD) were purchased from Invitrogen (USA). Native EGF and Hoechst 33258 were from Sigma-Aldrich (USA). Rabbit polyclonal anti-EGFR antibody (#2232) was from Cell Signaling Technology (USA), mouse monoclonal anti-EEA1 antibody (#610457) was from BD Transduction Lab (USA), mouse monoclonal anti-alpha-tubulin antibody (#T5168) was from Sigma (USA) and mouse monoclonal anti-Lamp-1 [H4A3] antibody (#ab25630) was from Abcam (USA). Alexa Fluor 568 goat anti-rabbit IgG, Alexa Fluor 488 goat anti-rabbit IgG and Alexa Fluor 488 goat anti-mouse IgG were from Invitrogen (USA). LysoTracker Green DND-26 (#L7526) was from Invitrogen (USA). Other chemicals were from Sigma-Aldrich unless otherwise stated.

### Cell culture

Human cervix epidermoid carcinoma HeLa cells and human epithelial lung carcinoma A549 cells (Russian Cell Culture Collection, Institute of Cytology RAS, St. Petersburg, Russia) were maintained in Dulbecco's modified Eagle medium (DMEM, Biolot, Russia) with 10% fetal bovine serum (FBS, Biolot, Russia) and 1% penicillin/streptomycin (GIBCO, USA) incubated at 37°C with 5% CO_2_. HeLa cells stably expressing GFP-α-tubulin (a kind gift of Dr. Mikitas’, Chumakov Institute of Poliomyelitis and Viral Encephalitides, RAMS, Moscow, Russia) were grown in DMEM/F12 containing 10% FBS, antibiotics and 500 ng/ml geneticin (G418) (Mediatechnic, USA). The cells were seeded in Lab-Tek borosilicate coverglass-bottomed chambered slides (Nunc) for live cell imaging or on Petri dishes with glass coverslips (Nunc). The cells were starved (0.1% FBS) overnight. Experiments were held at 60–70% confluent, 48 h after seeding.

### bEGF-savQD internalization into the cells

bEGF-savQD complexes were prepared *in vitro* in PBS by mixing of bEGF (2 nM) with savQDs (0.5 nM) for 30 min at 4°C [40]. EGF conjugation to QD did not affect emission spectrum and thus did not provoke additional aggregation (data not shown). The native EGF was added at 2 nM concentration.

The pulse-chase protocol was chosen to stimulate endocytosis under physiological conditions. The cells were washed twice with warm (37°C) DMEM and pulsed for 5 min with bEGF or bEGF-savQD at 37°C. Then, the unbound ligands were intensively washed out with warm DMEM and the cells were chased for the indicated time at 37°C before live cell imaging or fixation.

To study the endosome fusion, bEGF-savQDs with emitting wavelengths 525 and 655 nm were added sequentially. Initially, bEGF-savQD525 were added for 5 min at 37°C, then the cells were washed and chased for an additional 5- or 30-min intervals; then bEGF-savQD655 were added for 5 min at 37°C, the cells were washed and chased for 10, 60 and 120 min.

Although in the literature there are some data about apoptotic and cytotoxic effects of internalized QDs [62–64], in our experiments using a sufficiently low QDs concentrations (0.5 nM), we did not observe any apoptotic changes and cell death, even after prolonged incubation (48 h).

### Incubation with inhibitory conditions

Wortmannin (100 nM) was used to inhibit phosphoinositide 3-kinase. For this, the cells were incubated for 30 min with a drug. Afterwards, the cells were exposed to a solution containing the same drug concentration plus bEGF or bEGF-savQDs for 30 min.

### Compartment staining

For the vital staining of lysosomes and late endosomes, LysoTracker Green DND-26 was used at a concentration of 50 nM. After incubation with EGF-QD LysoTracker was added into the culture medium for 20 min prior to confocal imaging. For the vital staining of the nuclei, Hoechst 33258 was used at a concentration of 1.6 μM for 5 min.

### Immunofluorescent staining

The cells were fixed with 4% paraformaldehyde for 15 min, permeabilized with 0.5% Triton X-100 for 15 min and blocked with 1% BSA for 1 h. Fluorescence of bEGF-savQD was detected directly. To reveal EGFR localization the fixed cells were incubated with the primary anti-EGFR antibody (1:100) for 24 h at 4°C and then for 1 h with Alexa 568 or 488 goat anti-rabbit IgG (1:500). For co-localization analysis the cells were additionally incubated for 1 h at room temperature with primary antibodies of choice (anti-EEA1 antibody at 0.25 μg/ml concentration, anti-alpha-tubulin in 1:2000 and anti-Lamp-1 antibody in 1:100 dilutions) and for 1 h with the secondary antibodies (Alexa 488 goat anti-mouse IgG, 1:500). After immunostaining the cells were mounted into Fluorescent Mounting Medium (Dako Cytomation, Denmark).

### Confocal microscopy

The cells were examined with Leica TCS SP5 inverted laser scanning confocal microscope (Germany). For live cell imaging the cells seeded in Lab-Tek chambers and incubated with bEGF-savQD and vital dyes for the indicated time were analyzed using the temperature and gas control chamber (25°C and 5% CO_2_) of microscope. Serial images for video were taken at 37°C. QD525 and QD655 fluorescence was excited at 405 or 488 nm and registered in the 510–540 and 640–670 nm ranges, respectively. Alexa 488 and Alexa 568 were excited at 488 nm and 543 nm and registered in the 500–550 and 580–660 nm ranges, respectively. Hoechst 33258 and DAPI fluorescence was excited at 405 nm and registered in the 430–480 nm range. LysoTracker Green DND-26 was excited at 488 nm and registered in the 500–550 nm range. The specimens were observed with a ×40 oil immersion objective, followed by a 4 digital zoom magnification with an image size of 1024×1024 pixels. Images were taken in one or two spectral channels by sequential scanning mode, where only one laser was active at a time, to avoid spectral overlap. To optimize the signal to noise ratio, the final image was an average of three consequent runs. Z-series optical sections were taken at 0.5-μm intervals from the bottom to the top (14–16 sections). Images were acquired for at least 5 fields of view selected randomly per coverslip. Data were collected by Leica software as raw *.lif files and transferred as a series of tiff files for further analysis.

### Image and statistical analysis

All data were obtained from at least three independent experiments. In each experiment, 4–5 fields containing 15–20 cells totally were imaged for each time point. The images were processed and analyzed using Leica Confocal Software (Germany) and ImageJ software (National Institutes of Health, USA). In confocal images, the background of each channel was subtracted and, in some cases, brightness/contrast was adjusted only for presentation. No filter was applied in quantitative analyses. For presentation the most demonstrative single sections from the Z-stack were chosen. Then, the images were exported to Adobe Photoshop 5.0 for final processing.

The analysis of endosomes selected as the Region of Interest (ROI) by thresholding procedure in each cell was carried out using ImageJ. The number, integrated densities and perinuclear index of endosomes were calculated using ImageJ (menu command Analyze). Integrated density of endosome was evaluated by summarizing integrated intensities of all endosomes in a cell and normalizing them to the number of endosomes. Perinuclear index, defined as the ratio of the entire cell area to the area occupied by the bulk of endosomes, calculated for several time points, was used as a measure of the effectiveness of translocation of endosomes toward the microtubule organizing center. The quantitative co-localization analysis was performed using ImageJ JACoP Plugin [65] to determine Manders’ co-localization coefficient M1, which is defined as the sum of the intensities of the selected red objects containing green signal, divided by the sum of the intensities of all selected red objects. Thresholds were set by a visually estimated value for each channel. The surface plot displays a three-dimensional graph of the intensities of pixels and was plotted using ImageJ (menu command Analyze/Surface Plot). To study endosome translocations seria of images of a chosen cell was taken every 3 sec during 15 min in total. The endosomes that were in focal plane were individually tracked according to ImageJ Manual Tracking Plugin. The video of an endosome with typical behavior is presented in Supplement. Continuous track was created for an endosome that did not disappeared from focal plane at each frame of the temporal stack.

For all quantitative analyses, the results are presented as the mean ± 95% confidence interval for at least fifteen cells. The column charts were created using Microsoft Office Excel 2007.

## SUPPLEMENTARY MATERIALS FIGURES AND TABLES





## Abbreviations

QDs: Quantum dots
sav-QDs: strepavidin-conjugated QDs
EGF: epidermal growth factor
b-EGF: biotinilated EGF
EGFR: epidermal growth factor receptor
TK: tyrosine kinase
PI3P: phosphatidylinositol-3-phosphate
PI-3-kinase: phosphatidylinositol-3-kinase
EEA1: early endosome antigen 1
MVE: multivesicular endosome
GFP: green fluorescent protein
Lamp-1: lysosomal-associated membrane protein 1
