# Supplementary material for: EGF receptor lysosomal degradation is delayed in the cells stimulated with EGF-Quantum dot bioconjugate but earlier key events of endocytic degradative pathway are similar to that of native EGF
Source: Oncotarget. 2017 May 15;8(27):44335–50. doi: 10.18632/oncotarget.17873 (PMC5546484; doi:10.18632/oncotarget.17873)
Supplement: Supplementary file 1 [file oncotarget-08-44335-s001.pdf]

EGF receptor lysosomal degradation is delayed in the cells stimulated with EGF-Quantum dot bioconjugate but earlier key events of endocytic degradative pathway are similar to that of native EGF

Supplementary Materials

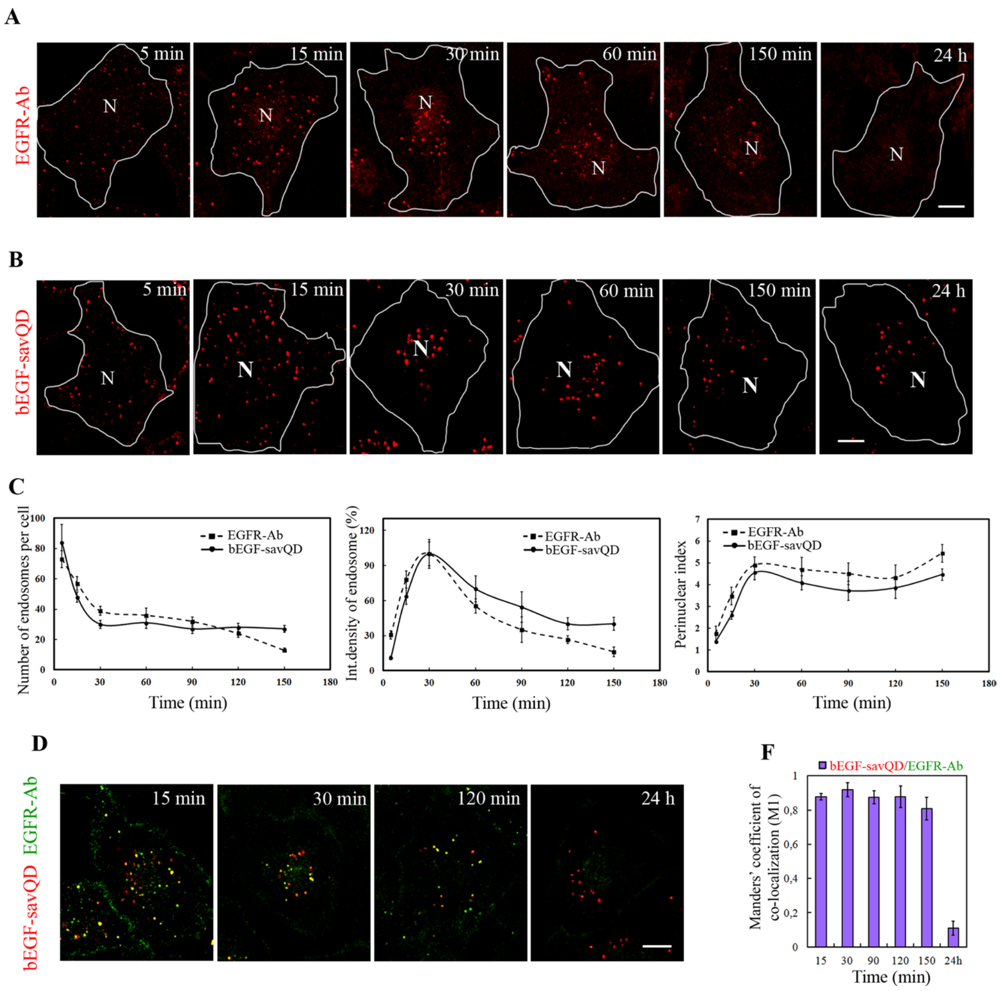

Supplementary Figure 1: Time-dependent imaging of EGFR and EGF-QDs distribution in A549 cells. A549 cells were incubated with (**A**) 2 nM EGF or (**B**) bEGF-savQD (2:0.5 nM) using pulse-chase protocol for the indicated time. (A) At each time point, the cells were fixed and immunostained with the anti-EGFR antibody (Alexa 568) before confocal microscopy. (B) For each time point a new well from the chambered cover glasses were used and images were taken in live cells without fixation. (**C**) The number of endosomes, their integrated densities and perinuclear index per cell (from the experiment described in A and B) were calculated for each time point using ImageJ. (**D**) A549 cells were incubated with bEGF-savQD (2:0.5 nM) using pulse-chase protocol for the indicated time. Then the cells were fixed and immunostained with the anti-EGFR antibody (Alexa 488) before confocal microscopy. Co-localizations between bEGF-savQD and EGFR-Ab were quantified using Manders' coefficient (M1). Each image is representative for the corresponding time point of at least three independent experiments. White lines in images outline cell boundaries and "N" marks the nucleus position. The data presented as the mean ± 95% confidence interval of three independent experiments. Scale bars: 10 µm.

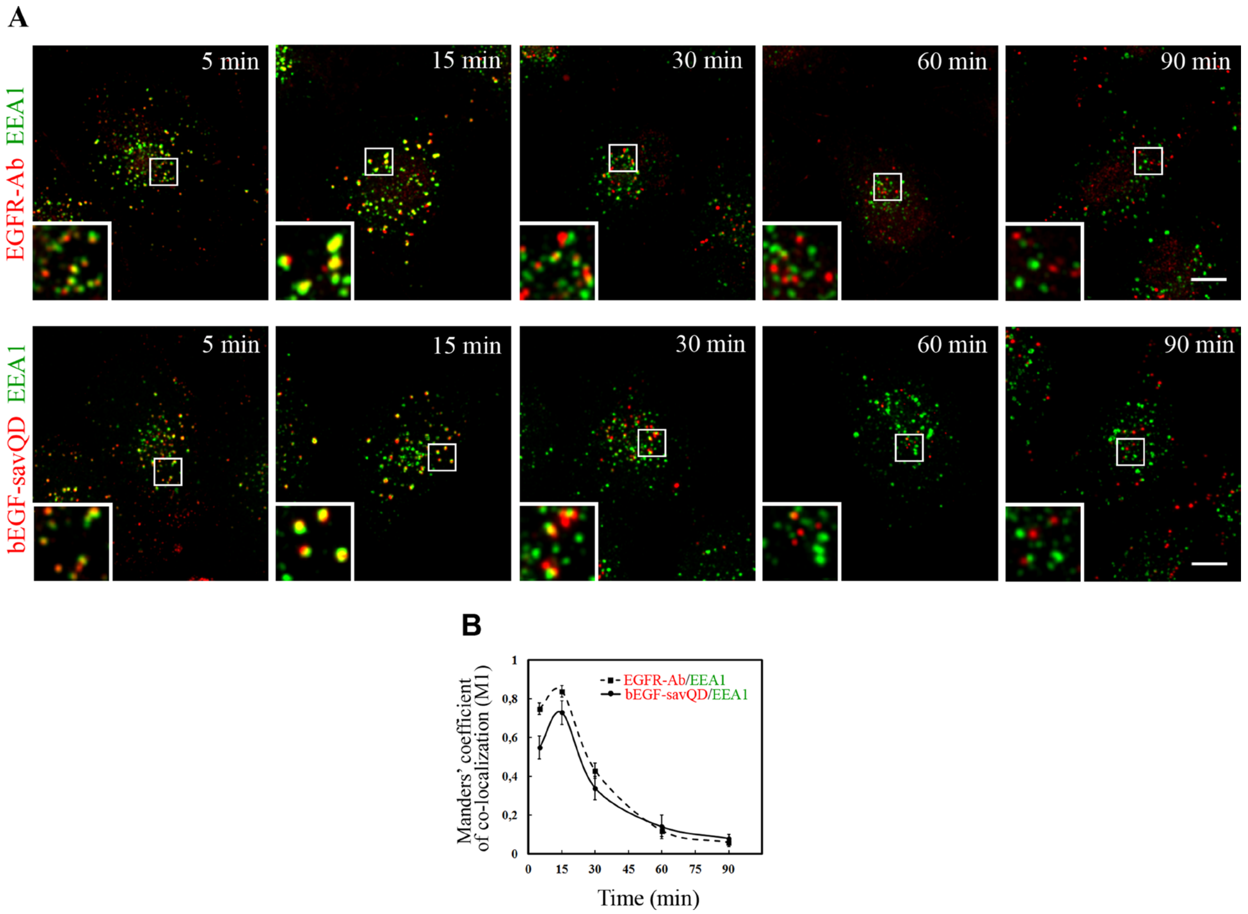

Supplementary Figure 2: Immuno-co-localization of EGFR and bEGF-savQD with EEA1. (**A**) A549 cells were incubated with 2 nM EGF or bEGF-savQD (2:0.5 nM) using pulse-chase protocol for the indicated time. Then cells were fixed and immunostained with the anti-EGFR (Alexa 568) and anti-EEA1 (Alexa 488) antibodies or in the case of bEGF-savQD cell with the anti-EEA1 (Alexa 488) antibody before confocal microscopy. The insets represent enlarged views (3 ×) of the corresponding boxed region of the cell. Each image is representative for the corresponding time point of at least three independent experiments. Scale bars: 10 µm. (**B**) Co-localizations between EGFR-Ab or bEGF-savQD and EEA1 were quantified using Manders' coefficient (M1). The data presented as the mean ± 95% confidence interval of three independent experiments.

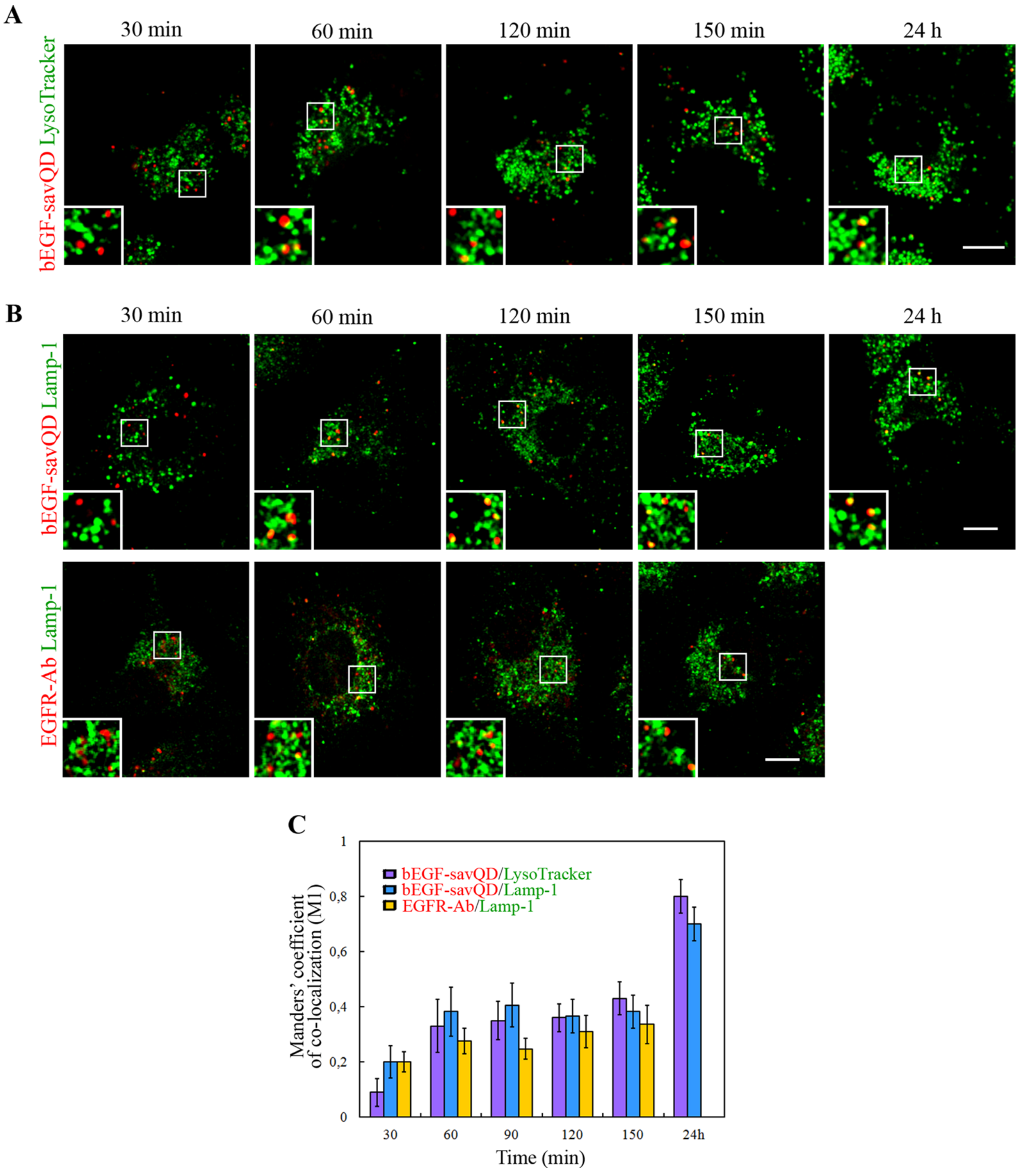

Supplementary Figure 3: Analysis of interaction of the endosomes containing bEGF-savQDs or EGFR with lysosomes. A549 cells were incubated with bEGF-savQD (2:0.5 nM) or 2 nM EGF using a pulse-chase protocol for the indicated time. (**A**) Live cells were analyzed and for each time point a new well from the chambered cover glasses were used. LysoTracker Green DND-26 was added into the culture medium for 20 min prior to confocal imaging. (**B**) At each time point the cells were fixed and immunostained with Lamp-1 (Alexa 488) or with anti-EGFR (Alexa 568) and Lamp-1 (Alexa 488) antibodies before confocal microscopy. The insets represent enlarged views (2.3 ×) of the corresponding boxed region of the cell. (**C**) Co-localizations between bEGF-savQD or EGFR-Ab and lysosomal markers were quantified using Manders' coefficient (M1). The data presented as the mean ± 95% confidence interval of three independent experiments. Each image is representative for the corresponding time point of at least three independent experiments. Scale bars: 10 µm.

Supplementary Video 1: Live cell imaging of QD-labeled endosome tracking in HeLa cells expressing GFP-α-tubulin. A representative time-lapse video demonstrates the typical behavior of the endosome bearing bEGF-savQDs. The first frame corresponds to 30 min time point after endocytosis stimulation by bEGF-savQD (2:0.5 nM). Here, 89 frames were taken every 3 sec until the endosome disappeared from focal plane. The movie was compressed to 5 frames per second. Scale bar: 2 µm. The trajectory of the center of visible endosome was plotted usind ImageJ Manual Tracking Plugin. See Supplementary_Video_1
